# The Src homology-2 protein Shb modulates focal adhesion kinase signaling in a *BCR-ABL* myeloproliferative disorder causing accelerated progression of disease

**DOI:** 10.1186/1756-8722-7-45

**Published:** 2014-06-21

**Authors:** Karin Gustafsson, Maria Jamalpour, Camilla Trinh, Michael G Kharas, Michael Welsh

**Affiliations:** 1 Department of Medical Cell Biology, Uppsala University, Husargatan 3, 75123 Uppsala, Sweden; 2 Molecular Pharmacology and Chemistry Program and Center for Cellular Engineering, Memorial Sloan Kettering Institute, 1275 York Ave, 10065 New York, NY, USA

**Keywords:** *BCR-ABL*, Focal adhesion kinase, Shb, Chronic myeloid leukemia, Neutrophilia

## Abstract

**Background:**

The Src homology-2 domain protein B (Shb) is an adapter protein operating downstream of several tyrosine kinase receptors and consequently Shb regulates various cellular responses. Absence of Shb was recently shown to reduce hematopoietic stem cell proliferation through activation of focal adhesion kinase (FAK) and thus we sought to investigate Shb’s role in the progression of leukemia.

**Methods:**

Wild type and *Shb* knockout bone marrow cells were transformed with a retroviral *BCR-ABL* construct and subsequently transplanted to wild type or *Shb* knockout recipients. Disease latency, bone marrow and peripheral blood cell characteristics, cytokine expression, signaling characteristics and colony formation were determined by flow cytometry, qPCR, western blotting and methylcellulose colony forming assays.

**Results:**

It was observed that *Shb* knockout *BCR-ABL*-transformed bone marrow cells produced a disease with death occurring at earlier time points compared with corresponding wild type controls due to elevated proliferation of transformed bone marrow cells. Moreover, significantly elevated interleukin-6 and granulocyte colony-stimulation factor mRNA levels were observed in *Shb* knockout c-Kit + leukemic bone marrow cells providing a plausible explanation for the concurrent peripheral blood neutrophilia. *Shb* knockout leukemic bone marrow cells also showed increased ability to form colonies in methylcellulose devoid of cytokines that was dependent on the concomitantly observed increased activity of FAK. Transplanting *BCR-ABL*-transformed *Shb* knockout bone marrow cells to *Shb* knockout recipients revealed decreased disease latency without neutrophilia, thus implicating the importance of niche-derived cues for the increase of blood granulocytes.

**Conclusions:**

Absence of *Shb* accelerates disease progression by exerting dual roles in *BCR-ABL*-induced leukemia: increased cell expansion due to elevated FAK activity and neutrophilia in peripheral blood, the latter dependent on the genetic background of the leukemic niche.

## Background

Hematopoiesis is a life-long process supported by a finely tuned network of proto-oncogenes and tumor suppressor genes controlling the self-renewal and proliferation of hematopoietic stem and progenitor cells (HSCs and HPCs). Deregulation of any of these elements has the potential to give rise to neoplasms [1]. Leukemic cells thus show characteristics of upregulated signaling cascades promoting self-renewal, increased cell cycle entry as well as prevention of apoptosis [2,3].

Chronic myeloid leukemia (CML) is myeloproliferative malignancy induced by the translocation between chromosomes 9 and 22 leading to the fusion of the *c-ABL* gene with the *break point cluster region (BCR)* gene [4]. The resulting oncogene *BCR–ABL* is a constitutively active tyrosine kinase with the ability to affect a broad range of signaling pathways including Ras, phosphatidylinositol-3 kinase (PI-3 K), and Rac [5-8]. Hence, cells expressing *BCR-ABL* display increased proliferative ability combined with reduced apoptotic rates and abnormal migratory characteristics [9-12]. *BCR-ABL* may, in addition, cause other types of leukemia.

Intracellular signaling events are not the only factors contributing to the progression of the disease. A common feature of most types of tumors is their ability to change the microenvironment to promote neoplastic growth. The tumor cells can either secrete tumor –promoting factors or the surrounding stroma can be induced to generate conditions favorable for expansion of leukemic cells [13,14]. CML bone marrow secretes increased levels of interleukin -6 (IL -6) and granulocyte colony –stimulating factor (G –CSF), both established as cytokines that stimulate myeloid expansion and differentiation [10,11,15-17]. Additionally, in leukemia, the stromal compartment has a reduced ability to support normal hematopoiesis, thus further enhancing the growth advantage of the leukemic cells [10,11,18,19].

The adaptor protein Shb is one of four members in a family of adaptor proteins with homologous tyrosine phosphorylation sites and Src homology 2 (SH2) domains [20-23]. Shb has been shown to operate downstream of tyrosine kinase receptors exerting versatile effects on a number of signaling pathways [24]. The SH2 domain of Shb binds to phosphotyrosines on activated receptors such as the platelet derived growth factor receptor (PDGFR), the IL-2 receptor and the T cell receptor (TCR) [24]. Shb’s various signaling domains further recruit intracellular signaling mediators, including focal adhesion kinase (FAK), Src, phosphatidylinositol 3-kinase (PI3K), Vav-1, and c-Abl [24,25], thereby regulating cytoskeletal rearrangements, proliferation as well as apoptosis [24].

Shb’s influence on the hematopoietic system has been documented in a number of studies. *Shb* knockout embryonic stem cells display reduced colony formation and delayed expression of hematopoietic markers [26]. CD4+ T_H_ cells isolated from a *Shb* knockout mouse exhibit a T_H_2 biased cytokine profile upon *in vitro* stimulation [27]. In HSCs, the loss of *Shb* results in hyperactivation of FAK leading to impaired HSC proliferation and failure to uphold long –term maintenance of the myeloid compartment [28]. The reduction of HSC proliferation prompted us to investigate Shb’s role in a stem cell mediated myeloproliferative model. *BCR-ABL*-induced myeloid disease is one of the most established systems to study factors that are known to be coordinated downstream of tyrosine kinase signaling. We observe that *Shb*-deficiency results in a more rapid progression of disease.

## Results

### Loss of Shb results in BCR-ABL-driven leukemogenesis with shorter latency

**Figure 1:**
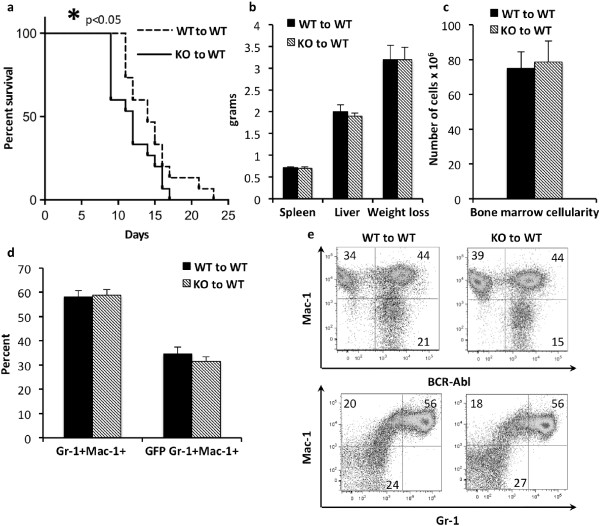
**Effects of Shb deletion on disease progression in murine model of CML. (a)** Kaplan-Meier curve demonstrating survival of mice receiving either wild type or *Shb* knockout *BCR-ABL* transformed bone marrow from 5-FU treated mice. **(b)** Analysis of various disease parameters including liver and spleen weight as well as weight loss at the end-stage of the disease. **(c)** Bone marrow cell numbers from the tibia, femur and the iliac bones were determined at the time of death. **(d and e)** Bone marrow cells were stained with fluorescently labeled antibodies directed against Gr-1 and Mac-1 and subsequently analyzed with FACS for GFP (*BCR-ABL*), Mac-1 and GR-1. Plots are representative of a typical experiment. Data are presented as mean ± SEM and based on 15 mice of each genotype from 5 independent experiments (retroviral transformation and transplantation occurring at 5 separate occasions). *denotes p < 0.05 as determined by Student’s *t*-test.

**Figure 2:**
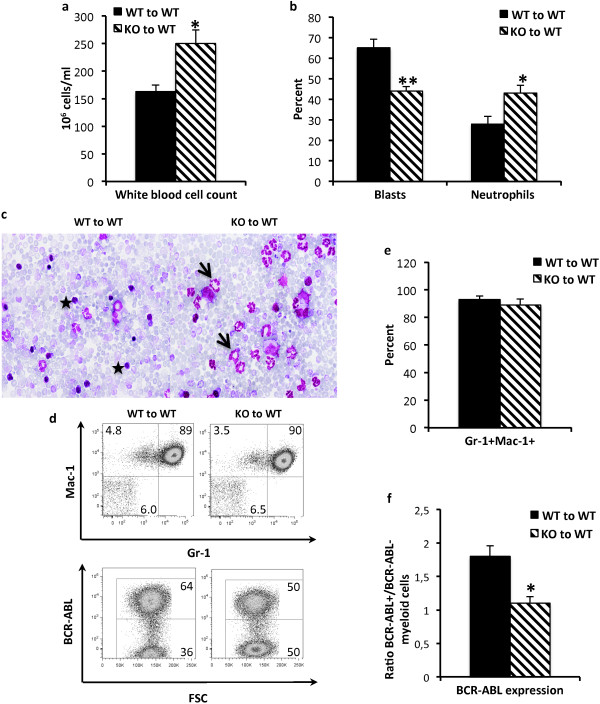
**Evaluation of peripheral blood profile in leukemic wild type and Shb knockout mice.** Peripheral blood smears were stained with May Grünwald-Giemsa; **(a and c)** the white blood cell count was established by differential counts and **(b and c)** the proportions of morphologically mature and immature cells were determined [stars point to blast cells and arrow to mature neutrophils]. **(d and e)** FACS analysis of Gr-1 and Mac-1 expression in peripheral blood. **(d and f)** The ratio of *BCR-ABL*^+^ and *BCR-ABL*^-^ within the myeloid compartment was determined by evaluation of GFP^+^ expression using FACS analysis. The plots are representative of a typical experiment. The results are presented as mean values ± SEM from 9 mice of each genotype in 3 independent experiments. ** and * represents p < 0.01 and p < 0.05 respectively as determined by Student’s *t*-test.

A recent study suggested that *Shb* knockout HSCs are less proliferative and fail to maintain the myeloid compartment over time [28] and consequently, we decided to investigate the effect of *Shb* deletion on the development of myeloid neoplasia. *Shb* knockout and wild type bone marrow cells were transformed with *BCR-ABL-GFP* encoding retrovirus and subsequently transplanted to wild type recipients. As the mice were monitored for disease progression *Shb* knockout recipients displayed symptoms and became moribund at earlier time points than their wild type counterparts (Figure 1a). In addition, when comparing the average life-span of wild type and *Shb* knockout transplanted mice in each individual experiment (a separate event of parallel wild type and *Shb* knockout bone marrow transfection followed by transplantation to recipient mice of which mean survivals were determined in each group) it was observed that absence of *Shb* shortened survival by 2.7 ± 0.4 days (n = 5, p < 0.01). Upon gross pathological examination there were no differences between mice receiving wild type or *Shb* knockout leukemic bone marrow at the time of death. Weight loss is one of the more prominent symptoms at the end stage of the disease; *Shb* null mice started losing weight earlier but the end stage weight did not significantly differ between the two groups (Figure 1b). The number of cells was also found to be equal in wild type and *Shb* knockout bone marrows (Figure 1c) and flow cytometric analysis of myeloid lineage markers Gr-1 and Mac-1 failed to demonstrate any differences between wild type and *Shb* deficient bone marrows in either GFP + (*BCR-ABL-GFP*) or total myeloid cell numbers (Figure 1d and e) [29]. As the leukemia progresses, immature hematopoietic cells will be driven out of the bone marrow and extramedullary hematopoiesis will occur at alternative sites such as spleen and liver. The result is considerable splenomegaly and hepatomegaly. In addition, leukemic cells collect in the lungs causing pulmonary hemorrhage [30]. End stage spleen and liver weights were, however, found to be similar for mice transplanted with wild type and *Shb* knockout bone marrows (Figure 1b) and hematoxylin-eosine stained sections of spleens, livers and lungs also did not reveal any differences with regards to leukemic cell infiltration (Additional file 1: Figure S1). The percentage GFP + myeloid cell number was decreased slightly in *Shb* knockout spleen whereas the non-leukemic myeloid cell percentage was unchanged (Additional file 1: Figure S1). Analysis of May Grünwald-Giemsa stained peripheral blood smears did on the other hand reveal significantly increased numbers of leukocytes (Figure 2a and c) in the absence of *Shb*. Normally, more aggressive forms of myeloid leukemia are distinguished by elevated levels of immature, blast cells in peripheral blood [30]. *Shb* deficiency did, however, confer increased proportions of cells with a mature neutrophil morphology and reduced relative numbers of blasts (Figure 2b and c). Although the relative proportion of blast cells was lower in the *Shb* knockout, absolute numbers of blast cells were not different (105 ± 11 × 10^6^ cells per ml in the wild type and 126 ± 22 × 10^6^ cells per ml in the *Shb* knockout). More mature stages of myeloid cells are defined as Gr-1^Hi^Mac-1^Hi^[29]. However Gr-1 and Mac-1 expression in peripheral blood did not differ significantly between wild type and *Shb* knockout samples (Figure 2d and e). Although the histological findings in peripheral blood could not be corroborated by changes in expression levels of lineage defining markers through FACS analysis, it is plausible that, as these cells are neoplastic, cell surface marker expression does not faithfully reflect morphological maturity. Notably, when the Gr-1^Hi^Mac-1^Hi^ population was examined for expression of *BCR-ABL*, determined by FACS analysis of GFP^+^ cells, recipients of *Shb* null transformed bone marrow displayed a decreased percentage of *BCR-ABL*^+^ myeloid cells in peripheral blood. *Shb* deficient samples showed an almost 1:1 ratio of *BCR-ABL*^+^ to *BCR-ABL*^-^ cells whereas wild type recipients had a ratio of 1.8:1 (Figure 2d and f). Since the total blood cell count was higher in the *Shb* knockout (Figure 2a), the absolute numbers of *BCR-ABL*^+^ myeloid cells did, however, not differ between wild type and knockout, suggesting that the non-malignant pool of myeloid cells had selectively expanded in the absence of *Shb* (Additional file 2: Figure S2).

In summary, the loss of *Shb* expression in malignant hematopoiesis exhibits a disease with shorter latency. Simultaneously, elevated numbers of mature neutrophil granulocytes are observed in peripheral blood.

### Shb deletion confers a proliferative advantage and reduces apoptosis in BCR-ABL^+^ lineage-negative cells

**Figure 3:**
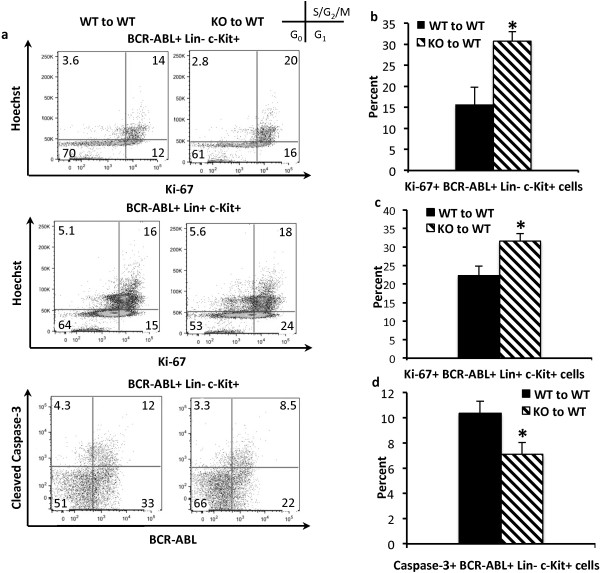
**Proliferation and apoptosis in BCR-ABL**^**+**^**hematopoietic progenitors assessed by flow cytometric analysis of Ki-67 and cleaved Caspase-3. (a, b and c)** The cell cycle status in *BCR-ABL*^+^Lin^-^c-Kit^+^ and *BCR-ABL*^+^Lin^+^ bone marrow was examined by staining for proliferation marker Ki-67 in combination with the DNA binding dye Hoechst 33342. FACS plots are representative of an average experiment. **(a and d)** The staining for presence of cleaved Caspase-3 was used to determine the percentage of apoptotic cells within the *BCR-ABL*^+^Lin^-^c-Kit^+^ population. FACS plots are representative of a typical experiment. Data are presented as mean values ± SEM with 9 mice from each genotype from 3 independent experiments. *denotes p < 0.05 as determined by Student’s *t*-test.

In order to further examine how the loss of *Shb* expression accelerates *BCR-ABL*-induced leukemia, the proliferation status of hematopoietic stem and progenitor cells was determined at the time of death. Most of the bone marrow’s stem and progenitor potential is found within the Lin^-^c-Kit^+^ population of cells. Flow cytometric analysis of the proliferation marker Ki-67 within the *BCR-ABL*^+^ Lin^-^ c-Kit^+^ population revealed a 1.9-fold increase in the proliferation rate (Figure 3a upper panel and Figure 3b) in *Shb* knockout bone marrow. Moreover, the more differentiated Lin^+^ c-Kit^+^ population was also found to exhibit an elevated cell cycle rate in *Shb* null bone marrow (Figure 3a middle panel and Figure 3c). Absence of *Shb* did not affect the sizes of the Lin^-^c-Kit^+^CD150^+^CD48^-^, the Lin^+^c-Kit^+^, and the Lin^-^c-Kit^+^ populations (Additional file 3: Figure S3a, b and c).

A hallmark of *BCR-ABL* transformed bone marrow cells is their resistance to apoptosis [9]. To determine if the apoptotic rate was also altered in the absence of *Shb*, *BCR-ABL*^+^ Lin^-^c-Kit^+^ bone marrow cells were stained for cleaved caspase-3 when the mice became moribund. A modest, but significant decrease in the percentage of cleaved caspase-3^+^ cells indicates that *BCR-ABL*^+^ Lin^-^ c-Kit^+^ cells are less apoptotic as a result of *Shb* deletion (Figure 3a lower panel and Figure 3d). There was no effect on apoptosis in the Lin^+^ c-Kit^+^ population (data not shown). In conclusion, the data imply that ablation of *Shb* expression is associated with an increased expansion of the leukemic lineage-negative progenitor cell population through enhancement of proliferation and survival.

### Shb knockout leukemic bone marrow is hypersensitive to cytokine stimulation and expresses increased levels of G-CSF and IL-6

**Figure 4:**
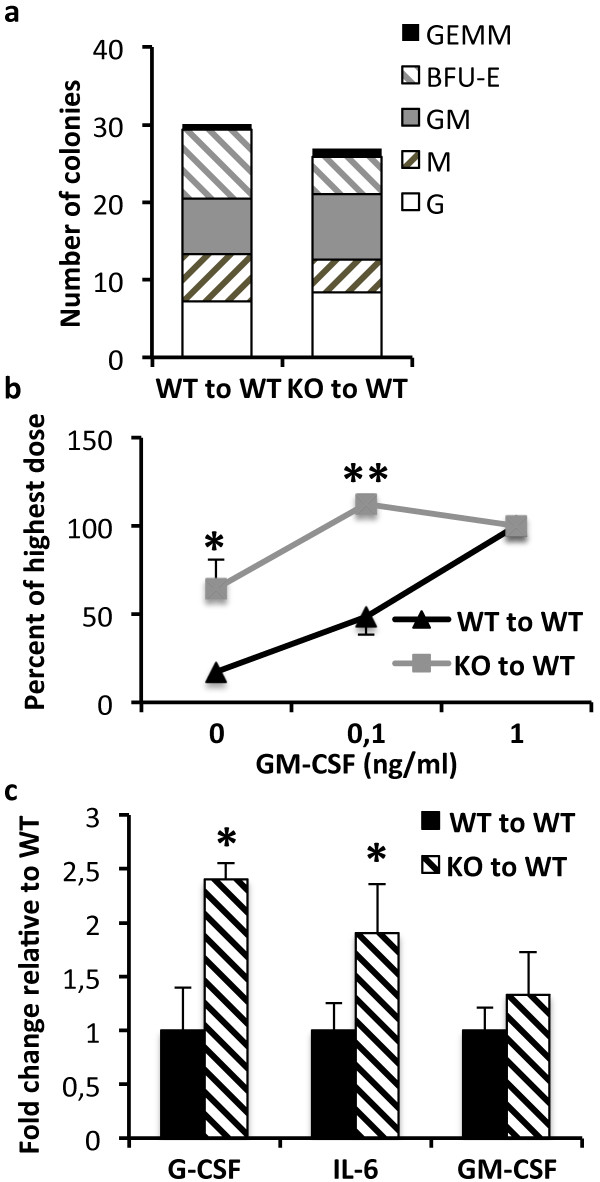
**Effects of Shb deficiency on hematopoietic colony formation ability and cytokine expression in BCR-ABL-transformed bone marrow. (a)** Bone marrow cells were plated on M3434 semisolid medium containing cytokines supportive of myeloid and erythroid colony growth. The number and types of colonies were determined on day 10 of culture [Granulocyte Erythroid Monocyte Megakaryocyte (GEMM), Burst-forming Unit Erythroid (BFU-E), Granulocyte Monocyte (GM), Monocyte (M), Granulocyte (G)]. Data are means based on 3 mice of each genotype. **(b)** A gradient of 0, 0.1 and 1 ng/ml of GM-CSF was used to evaluate cytokine responsiveness in leukemic bone marrow cells. Results are presented as percentage of the highest GM-CSF dose. Means ± SEM are representative of 3 mice of each genotype. **(c)** The expression levels of various hematopoietic cytokines were determined by semi-quantitative real-time RT-PCR in samples isolated from c-Kit^+^ leukemic bone marrow. All C_t_ values were normalized to β-actin and *Shb* knockout samples were related to corresponding wild type values. Means are presented as 2^-ΔC^_t_ ± SEM to demonstrate fold change in mRNA content. Data are based on 6 mice of each genotype from 2 independent experiments for c-Kit^+^ cells and 3 mice of each genotype from 1 experiment for unfractioned bone marrow. *denotes p < 0.05 respectively as determined by Student’s *t*-test.

Normal bone marrow only forms hematopoietic colonies in the presence of the appropriate cytokines; malignantly transformed stem and progenitor cells however, are capable of growth factor independent proliferation and differentiation [31]. To assess the response to cytokine stimulation, cells from leukemic wild type and *Shb* knockout bone marrows were seeded into semisolid media containing a cytokine cocktail with the potential to support myeloid as well as erythroid differentiation. Both wild type and *Shb* null bone marrow readily formed colonies with no significant difference in the number or the types of colonies formed (Figure 4a). When plating the cells on a gradient of granulocyte-monocyte colony-stimulating factor (GM-CSF), noticeable differences were observed as compared to the experiments in which the cells were plated in methylcellulose supplemented with multiple cytokines. It was revealed that *Shb* deficient bone marrow had an increased potential to support cytokine-independent growth and responded at lower concentrations of GM-CSF. In the absence of cytokines *Shb* knockout bone marrow formed 3 times as many colonies as the wild type (Figure 4b) and at a concentration of 0.1 ng/ml GM-CSF the knockout produced twice as many colonies.

To further explore the cytokine signaling networks in our model, the expression levels of a number of cytokines, known to regulate *BCR-ABL*-induced leukemogenesis and hematopoietic cell proliferation and differentiation, were determined. In order to facilitate a distinction between cytokine production within the bone marrow as a whole and the hematopoietic compartment, bone marrow was first fractionated based on c-Kit expression, as most HSCs and HPCs are c-Kit^+^. Transcription of GM-CSF was unchanged in both the c-Kit^+^ compartment and in unfractionated bone marrow (Figure 4c and Additional file 4: Figure S4). The expression of G-CSF and IL-6 was on the other hand significantly elevated in c-Kit^+^ cells (Figure 4c) but not in total bone marrow (Additional file 4: Figure S4), suggesting that the increased cytokine production is limited to the hematopoietic compartment. This is in line with previous reports suggesting that leukemic progenitors secrete IL-6 and G-CSF in an autocrine fashion [10,32]. Other factors, such as TNFα, IL-1α, IL-1β, IL-4, MIP-1α, MIP-1β, SCF, IL-3, thrombopoietin and angiopoietin-2, have also been demonstrated to be important factors in promoting the proliferation of leukemic HSCs and HPCs [10,18]. The transcript levels of these factors were therefore determined in c-Kit enriched and unfractionated bone marrow, but no differences were detected between wild type and *Shb* deficient samples (Additional file 4: Figure S4).

Additionally, G-CSF has been linked to impaired bone marrow retention of leukemic HSCs due to decreased production of the chemokine CXCL12 by bone marrow stromal cells [10]. There was however no detectable difference in CXCL12 expression in total (unfractionated) bone marrow (relative expression of CXCL12 was 1 ± 0.15 in wild type and 0.73 ± 0.25 in *Shb* knockout). Reduced expression of CXCL12 in bone marrow has been shown to increase the number of HSCs found in the spleen [10]. Flow cytometric analysis of splenic HSCs revealed no difference between wild type and *Shb* knockout recipients (data not shown), further supporting the notion that the elevated levels of G-CSF do not appear to affect the invasion of *Shb* deficient leukemic HSCs to the spleen.

These results thereby indicate that *Shb* serves as a modulator of cytokine expression levels thus possibly explaining the increased numbers of neutrophils in blood from *BCR-ABL* transformed *Shb* deficient bone marrow.

### Accelerated BCR-ABL-induced leukemia in Shb knockout recipient mice as a consequence of Shb deficiency

**Figure 5:**
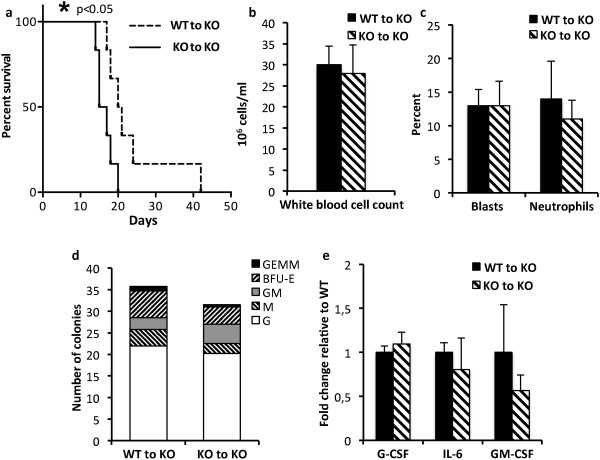
**Effects of Shb deletion on disease progression of BCR-ABL-transformed bone marrow cells transplanted to Shb knockout recipient mice. (a)** Kaplan-Meier curve demonstrating survival of *Shb* knockout mice receiving either wild type or *Shb* knockout *BCR-ABL*-transformed bone marrow. **(b)** White blood cell counts in peripheral blood at the time of death. **(c)** Relative numbers of mature neutrophils and blasts in peripheral blood at the time of death. **(d)** Colony formation assay of c-Kit enriched bone marrow cells grown in cytokine-supplemented methylcellulose. **(e)** Cytokine mRNA levels in c-kit-enriched bone marrow cells.

We decided to investigate the progression of disease when *BCR-ABL*-transformed wild type and *Shb* knockout bone marrow cells were transplanted to *Shb* knockout recipient mice instead. The myeloproliferative leukemia was also accelerated in this setting when compared to wild type control (Figure 5a). Transplantation of *BCR-ABL*-transfected bone marrow to wild type and knockout recipients in a parallel experiment revealed no consistent difference in latency (results not shown). Unlike the situation with wild type recipients (see Figure 2b and c), no signs of neutrophilia were observed (Figure 5b and c) when *Shb* knockout bone marrow was transplanted to knockout recipients. Colony formation in methylcellulose supplemented with cytokines was similar regardless of bone marrow cell genotype (Figure 5d). The expression of G-CSF and IL-6 in c-Kit^+^ bone marrow cells was unaltered by *Shb* deficiency (Figure 5e), explaining the absence of peripheral blood neutrophilia. This contrasted to what was seen with wild type recipients, suggesting that the host genotype in combination with the genotype of the transformed donor cells is critical for this response. The most likely explanation is interplay between transformed bone marrow cells and leukemic niche cells. Consequently, increased production of G-CSF and IL-6 in *BCR-ABL Shb* knockout bone marrow cells required a wild type niche.

### Enhanced FAK and STAT3 activity of Shb deficient leukemic c-Kit^+^ bone marrow cells

**Figure 6:**
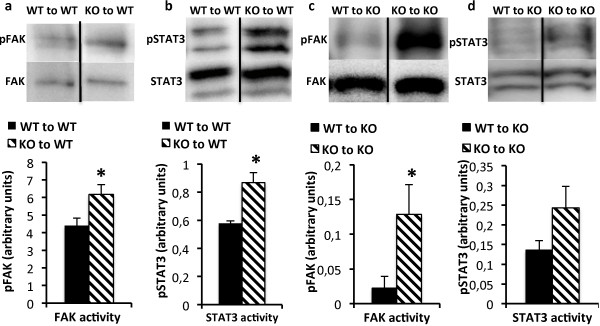
**Activity of FAK (a, c) and STAT3 (b, d) in c-Kit**^**+**^**bone marrow cells from leukemic mice.** The phosphorylation status of FAK and STAT3 was determined by Western blot analysis. **(a and b)** show bone marrow cells from wild-type recipients, **(c and d)** show bone marrow cells from *Shb* knockout recipients. Activation was evaluated by immunoblotting for phospho- and total FAK and STAT3 respectively. Protein phosphorylation was related to total protein content on the same blot and signal strength was estimated by densitometric analysis. Means are presented in arbitrary units ± SEM and are based on 6 mice of each genotype in 2 independent experiments. * denotes p < 0.05 respectively as determined by Student’s *t*-test.

An increase in basal signaling activity in response to ablated *Shb* expression has been noted in several types of tissue [27,28]. The activities of pathways controlling proliferation and survival were therefore analyzed in unstimulated c-Kit^+^ bone marrow cells. As noted in non-malignant HSCs [28], FAK activity was also increased in leukemic *Shb* knockout c-Kit^+^ bone marrow (Figure 6a) transplanted to wild type mice. Myeloid leukemias are often characterized by hyperphosphorylation of STAT3 and STAT5 [33]. STAT5 phosphorylation was similar in *Shb* knockout and wild type samples (Additional file 5: Figure S5), whereas STAT3 activity was significantly increased in the knockout (Figure 6b). Notably, both G-CSF and IL-6 signal via STAT3 activation [34,35]. When FAK and STAT3 activities were monitored in leukemic *Shb* deficient c-Kit^+^ bone marrow isolated from *Shb* knockout recipient mice, increased activity of FAK was detected (Figure 6c) whereas STAT3 activity was not consistently elevated (Figure 6d). Consequently, FAK activation is a consistent feature of *BCR-ABL*-transformed *Shb* knockout bone marrow cells. STAT3 activity, on the other hand reflects the local cytokine milieu, which in turn depends on the interactions between the niche and the transformed bone marrow cells.

### Elevated FAK activity is important for the proliferative ability of BCR-ABL-transformed Shb knockout bone marrow cells

**Figure 7:**
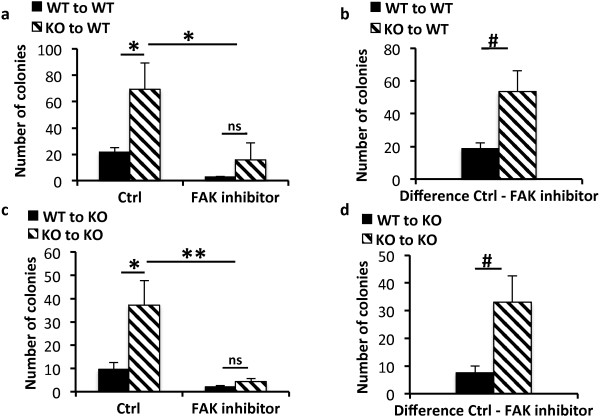
**Effect of FAK inhibition on colony formation of wild-type and Shb knockout BCR-ABL-transformed bone marrow cells.** c-Kit-enriched bone marrow cell (10^4^) were plated in methylcellulose in the absence of cytokines in the absence or presence of 10 μM FAK inhibitor 14 and cultured for 5 days after which colony numbers were determined. **(a)** Colony formation of bone marrow cells isolated from wild-type recipients. **(b)** Inhibition of colony formation by inhibitor on bone marrow cells isolated from wild-type recipients. **(c)** Colony formation of bone marrow cells isolated from *Shb* knockout recipients. **(d)** Inhibition of colony formation by inhibitor on bone marrow cells isolated from *Shb* knockout recipients. The values in (b) and (d) were obtained by subtracting the inhibitor values from the corresponding control values. Means ± SEM for 6 dishes (three mice) each group are given. * and ** indicate p < 0.05 and 0.01, respectively with one way ANOVA (Bonferroni). # indicates p < 0.05 when compared with wild-type using a Students’ *t*-test. Ns = not statistically significant.

To address the relevance of the elevated FAK activity, c-Kit^+^ leukemic bone marrow cells were isolated and cultured in methylcellulose without cytokine addition (*i.e.* absence of GM-CSF) in the absence (control) or presence of FAK inhibitor 14. *Shb* knockout bone marrow cell colony formation was increased compared with corresponding wild type controls regardless of whether the cells were isolated from wild type or *Shb* knockout recipient mice (Figure 7a and c). The FAK inhibitor caused a statistically significant reduction in the numbers of colonies formed by the *Shb* knockout bone marrow cells (Figure 7a and c). Dividing the number of colonies without FAK inhibitor treatment by that with FAK inhibitor treatment in the wild type situation yielded a ratio as high or even higher than the corresponding knockout situation, suggesting the possibility that there is indeed an effect of the FAK inhibitor on wild type leukemic bone marrow colony formation as well. However, the total number of colonies was too small relative the errors to obtain a statistical significant difference. We also determined the inhibitor-induced reduction in colony formation after FAK inhibition (Figure 7b and d) to illustrate the effect in the Shb knockout situation. This was significantly larger than that observed in wild type bone marrow cells (Figure 7b and d) suggesting a preferentially greater importance of FAK signaling for proliferation in transformed *Shb* knockout bone marrow cells. Assuming that the total number of colonies formed reflects the proliferative status of the bone marrows at the time of death, the data thus support the view that elevated FAK activity as a consequence of *Shb* deficiency causes increased cell expansion accelerating the progression of disease. It is also possible that FAK plays a role for wild type leukemic cell colony formation as well.

## Discussion

The adaptor protein Shb has been implicated in intracellular signaling events regulating proliferation, apoptosis and differentiation in a number of different cell types. Recently, HSC exhibited less proliferative activity in *Shb* knockout mice due to alterations in FAK signaling [28]. The present study aimed at relating those observations to conditions of neoplastic hematopoiesis. The results suggest that *Shb* depletion accelerates *BCR-ABL*-driven progression of myeloid neoplasia by causing decreased latency and increased numbers of myeloid cells in peripheral blood due to elevated FAK activity. In addition, niche-dependent cues have an impact on disease progression with a bearing on cytokine production in bone marrow cells and neutrophilia in peripheral blood.

FAK activity was recently noted to be significantly increased in normal HSCs in *Shb* deficient mice [28]. A similar increase in FAK activity was observed in *BCR-ABL*-transformed *Shb* knockout c-Kit^+^ bone marrow. Our current knowledge of FAK’s effects on leukemogenesis is limited although the bulk of data suggest that active FAK promotes leukemogenesis. *BCR-ABL* appears to induce FAK phosphorylation and siRNA silencing of FAK reduces survival of AML leukemia cell lines [36,37]. Additionally, observations from murine models and AML patients suggest that FAK may influence leukemia progression [38,39]. FAK is mainly activated by integrins, thus mediating signals between cells and their respective surroundings. Leukemic cells enhance their own growth by altering the bone marrow microenvironment [10,11,18]. FAK signaling could provide a key link between leukemic cells and the stromal cells of the hematopoietic niche. FAK expressing AML cells have been demonstrated to enhance the ability of bone marrow stroma to support leukemic growth through direct contact [40]. Moreover, in a recent study utilizing a murine model of *BCR-ABL*-induced leukemia, it was shown that soluble factors synergize with an unidentified contact-dependent mechanism to drastically change the hematopoietic niche composition to promote neoplastic progression [18]. The present colony formation experiments adding a FAK inhibitor in the absence of cytokines lend strong support to the notion that the increased tumor burden of transformed *Shb* knockout bone marrow cells is mainly due to elevated FAK activity. In *Shb* deficient *BCR-ABL*-transformed bone marrow, immature lin^-^c-Kit^+^ HSCs and HPCs as well as more differentiated Lin^+^ c-Kit^+^ cells, were found to proliferate at an increased rate and this finding is likely to reflect the same mechanism as the colony formation assay.

The *Shb* deficient myelodysplastic neoplasia phenotype is also intriguing as the rapid progression of disease is associated with a pronounced neutrophilia when studied in wild type recipient mice. In most cases, more aggressive forms of leukemia are distinguished by a blood profile dominated by immature blasts [30]. *Shb* deficient c-Kit^+^ leukemic bone marrow cells were on the other hand found to express increased levels of granulopoietic factors IL-6 and G-CSF in these experiments. Myeloid differentiation is supported by IL-6 through cell cycle regulation of myeloid progenitors and IL-6 also blocks lymphoid lineage commitment thus further enhancing myeloid expansion [16,41,42]. Moreover, leukemia progression is significantly hampered in *IL-6* knockout mice directly linking this cytokine to disease [11]. This may involve effects on both malignant and non-malignant cells [11]. Peripheral blood from recipients transplanted with transformed *Shb* knockout cells contained elevated numbers of *BCR-ABL*^-^ myeloid cells indicative of an expansion of non-malignant cells, possibly a result of the increased IL-6 expression. Neutrophil differentiation under homeostatic and stress conditions depends on G-CSF providing survival and differentiation signals to granulocytic progenitors [15,17]. Further demonstrating G-CSF’s effect on neutrophil expansion is the finding that chronic neutrophilic leukemia, a rare myeloproliferative disorder characterized by excessive expansion of the neutrophilic population in blood and bone marrow, is linked to activating mutations in the *CSF3R* gene, the human receptor for G-CSF [43].

STAT3 phosphorylation was significantly augmented in c-Kit^+^ bone marrow cells isolated from *Shb* knockout recipients on wild type background. Notably, G-CSF and IL-6 signaling pathways converge in the activation of the transcription factor STAT3 and STAT3 is the main mediator of the proliferation and survival signals provided by G-CSF and IL-6 [34,35,44,45]. The hyperphosphorylation displayed by *Shb* null cells is thus probably a result of the increased production of G-CSF and IL-6.

## Conclusions

The data presented suggest that Shb regulates cues in neoplastic bone marrow of importance for leukemic progression and that absence of *Shb* decreases disease latency. The *Shb-*dependent effects include bone marrow cell intrinsic pathways (FAK) as well as niche-dependent signals (cytokine production). Both of these components are considered as druggable targets. Further exploration of the effects of *Shb* deletion in hematopoietic malignancies is therefore of importance to increase our understanding of mechanisms that control leukemogenesis.

## Methods

### Mice

The generation of Shb knockout mice has been described previously [46]. The *Shb* knockout genotype is not viable on the C57Bl/6 background and the animals were therefore maintained on the Balb/c strain. The local animal ethics committee at Uppsala University approved all experiments.

### Bone marrow transduction and transplantation assay

The pMIG-*p210bcr/abl* vector was used to produce retroviruses directing the expression of *BCR-ABL-GFP* as described previously [47]. Balb/c *Shb* wild type or knockout mice 8-10 weeks old were treated with 5 –fluorouracil (5-FU) (Sigma-Aldrich, St. Lois, MO) at a dose of 150 mg/kg body weight 6 days prior to bone marrow isolation, in order to enrich for HSCs. Isolated donor bone marrow was stimulated in RPMI 1640 (Sigma Aldrich) supplemented with 10% FCS (Sigma Aldrich), 2 mM L-glutamine, streptomycin (0.1 mg/ml), penicillin (100 U/ml) (All from Gibco, Paisley, UK), IL-3 (10 ng/ml), stem cell factor (SCF) (10 ng/ml) and IL-6 (10 ng/ml) (all cytokines were purchased from PeproTech, Rocky Hill, NJ) for 24 hours. The cells were subsequently subjected to two rounds of spin infections over the following 48 hours as described previously [48]. Briefly, 8 × 10^6^ cells were centrifuged for 90 minutes at 1000 *g* and 30°C in medium containing the aforementioned supplements as well as 25% viral supernatant, 7.5 mM Hepes (Gibco) and 8 μg/ml Polybrene (Millipore, Watford, UK). Infection efficiency was determined following the second spin inoculation and just prior to transplantation by flow cytometric analysis of green fluorescent protein (GFP) expression on a FACSCalibur (BD Bioscience, Erembodegem, Belgium) and no differences were found between wild type and *Shb* knockout bone marrow (5.6 ± 1.0% in wt; 4.9 ± 1.1% in knockout). Recipient wild type or *Shb* knockout mice were irradiated with two doses of 4.5 Gy separated by at least 2 hours in a Nordion Gammacell 40 Exacto ^137^Cs irradiator (MDS Nordion, Ottawa, ON). Immediately following the second irradiation the recipients were retroorbitally injected with a dose of 0.4 – 1 × 10^6^ cells (equal number of wild type and *Shb* knockout cells was given per recipient mouse in each experiment, *i.e.* transfection/transplantation event).

### Pathological examination of diseased mice

The mice were monitored daily from day 6 post –transplantation for signs of disease such as a weight loss of more than 15% of initial body weight, lethargy and splenomegaly. Moribund mice were then sacrificed. Blood was collected immediately prior to sacrifice and samples were prepared for blood smears. Spleens, livers, and lungs were fixed in 4% buffered formalin and embedded in paraffin for later histopathological analysis. Iliac bones, femurs and tibias were dissected and bone marrow was collected. Bone marrow cells were extracted from the bones and used for further downstream applications. Single cell suspensions of spleen and bone marrow were also fixed in 4% paraformaldehyde to enable FACS analysis at a later time point.

### Histology

Fixed and paraffin embedded organs were sectioned in 5 μm sections, mounted on microscope slides (Menzel Gläser, Braunschweig, Germany) and stained with Hematoxylin –Eosin. For differential blood counts, peripheral blood smears were stained with May –Grünwald Giemsa.

### Fluorescent activated cell-sorting (FACS) analysis

Paraformaldehyde fixed peripheral blood and bone marrow single cell suspensions were stained with antibodies directed against Gr -1-PE (eBioscience, Hartfield, UK) and rat anti mouse Mac-1 (Invitrogen, Carlsbad, CA) followed by incubation with goat anti-rat PE-Cy5.5 (Invitrogen) secondary antibody.

In order to identify the HSC population, bone marrow and spleen cells were stained with a lineage excluding cocktail consisting of rat anti-mouse antibodies CD3, CD8, CD4, B220, CD19, Gr-1, and Mac-1. The samples were thereafter incubated with goat anti-rat PE-Cy5.5, followed by staining with CD150-PE-Cy7 (BioLegend, San Diego, CA), c-Kit-APC eFluor 780, and CD48-PE (eBioscience).

Proliferative and apoptotic rates were determined by analysis of Ki-67 and cleaved Caspase-3. Paraformaldehyde fixed bone marrow was stained for lineage defining markers and c-Kit as described above. This was followed by permeabilization with BD Cytoperm Buffer (BD Bioscience) and incubation with either Ki-67-PE antibody (BD Bioscience) or cleaved Caspase-3 antibody (Cell Signaling Technology, Beverly, MA). Cleaved Caspase-3 activity was detected by a PE-conjugated donkey anti-rabbit antibody (eBioscience).

All flow cytometric experiments were analyzed with a LSR II (BD Bioscience) and the data was analyzed with FlowJo (TreeStar, Ashland, OR).

### Colony forming assays

Freshly isolated bone marrow and spleen cells were plated on methylcellulose medium M3434 (Stem Cell Technologies, Vancouver, BC) at seeding densities of 2 × 10^4^ and 1 × 10^5^ cells, respectively. Bone marrow cells were also seeded onto M3231 supplemented with a gradient of GM-CSF (PeproTech) at concentrations of 0, 0.1 and 1 ng/ml or 10 μM FAK inhibitor 14 (Tocris Bioscience, Bristol, UK). Colonies were scored at day 10.

### RNA isolation and real-time reverse transcription PCR

In order to enrich bone marrow for hematopoietic stem and progenitor cells, c-Kit^+^ cells were isolated by magnetic separation with anti-c-Kit labeled magnetic microbeads (Miltenyi Biotec, Bergisch Gladbach, Germany), following the instructions provided by the manufacturer. The number of c-Kit^+^ cells was determined and RNA was subsequently isolated using a RNAeasy mini kit (Qiagen, Solna, Sweden). Analysis of gene expression was performed with one- step real-time reverse transcription PCR using QuantiTect™ SYBR® Green RT-PCR kit (Qiagen). The following PCR conditions were used; reverse transcription at 50°C for 20 minutes, inactivation at 95°C for 15 minutes, 50 cycles of denaturation at 94°C for 15 s, annealing for 25 s at 60°C, and extension at 72°C for 15 s. All primer sequences can be provided upon request. The PCR reactions were all run a LightCycler™ real- time PCR machine (Roche Diagnostics, Basel, Switzerland). The Cycle threshold (C_T_) values were estimated with the LightCycler Software v 4.1 and transcript levels were normalized by subtracting the corresponding β –actin values. Control was set at one differences and presented as 2^-ΔKOCt-WTCt^.

### Immunoblotting

Bone marrow samples were enriched for c-Kit^+^ cells as described above. Promptly after isolation cells were allowed to rest for 1 hour at 37°C in RPMI 1640 medium supplemented with 10% serum. The cells were subsequently lyzed in SDS sample buffer (250 mM Tris-HCl pH 6.8, 4% SDS, 10% glycerol, 0.006% bromophenol blue, 2% β-mercaptoethanol). Samples were then separated by SDS-PAGE and transferred to a Hybond-P membrane (GE Healthcare, Uppsala, Sweden). Blocking of the membranes were done over night at 4°C in 5% BSA, followed by probing for phospho-STAT3, STAT3, FAK (all from Cell Signaling Technology), and phospho-FAK (Invitrogen).

### Statistical analysis

All values are presented as mean ± SEM. Comparisons of two groups were analyzed by an unpaired Student *t*-test as all data sets were found to be normally distributed. For comparisons between multiple groups one-way ANOVA was used, followed by post hoc analysis with Bonferroni’s test. Statistical significance was set to p < 0.05.

## Abbreviations

FAK: Focal adhesion kinase; Shb: Src homology-2 domain protein B; IL: Interleukin; G-CSF: Granulocyte colony-stimulation factor; STAT: Signal-transducer and activator of transcription; FACS: Fluorescence-activated cell sorting; CML: Chronic myeloid leukemia; HSC: Hematopoietic stem cell; HPC: Hematopoietic progenitor cell; BM: Bone marrow; TNF: Tumor necrosis factor; SCF: Stem cell factor; MIP: Macrophage inflammatory protein; CXCL: Chemokine (C-X-C motif) ligand; GFP: Green fluorescent protein; BCR: Breakpoint cluster region.

## Competing interests

The authors declare that they have no competing interests.

## Authors’ contributions

KG, MK and MW conceived the experimental design. KG, MJ, CT and MW performed the experiments and analyzed the data. KG, MK and MW interpreted the data. KG and MW wrote the paper. All authors agree on its content.

## Supplementary Material

Additional file 1: Figure S1 A) Hematoxylin-eosin stained sections of lung, liver and spleen of diseased mice transplanted with *BCR-ABL* transformed bone marrow cells of wild type or *Shb* knockout background to wild type recipients. B) Percentage myeloid GFP + and GFP- cells in spleen. Means ± SEM are given and *indicates p < 0.05. Click here for file

Additional file 2: Figure S2 Absolute numbers of *BCR-ABL*^+^GFP^+^ and *BCR-ABL*^-^GFP^-^ myeloid cells in peripheral blood. GFP^+^ GR-1^Hi^ Mac-1^Hi^ and GFP^-^ GR-1^Hi^ Mac-1^Hi^ cells were identified with FACS analysis in order to estimate the number of *BCR-ABL* carrying cells. Means are presented in arbitrary units ± SEM and are based on 6 mice of each genotype in 2 independent experiments. *denotes p < 0.05 as determined by Student’s *t*-test. Click here for file

Additional file 3: Figure S3 Estimation of HSC proportions in murine *BCR-ABL* transformed bone marrow. The proportions of HSCs were established using FACS based on expression of lineage defining markers, c-Kit, CD48 and CD150 (a). Right panels are *Shb* knockout. (b) Percentage CD150+/CD48- cells among c-Kit+/Lin- cells and fraction of GFP + cells among the CD150+/CD48- cells. Means ± SEM for 6 experiments are shown. (c) Percentage lineage-/c-Kit + or lineage+/c-Kit + cells in bone marrows of wild type or *Shb* knockout *BCR-ABL* transformed bone marrow cells transplanted to wild type recipients. Means ± SEM for 6 mice of each genotype are given. Click here for file

Additional file 4: Figure S4 Evaluation of cytokine expression in c-Kit enriched and unfractionated (total) bone marrow. (a) Transcript levels of SCF, IL-3, thrombopoietin (Thpo) and angiopoietin-2 (Angpt2) were evaluated with semi-quantitative real-time RT-PCR using mRNA isolated from c-Kit + leukemic bone marrow samples. The expression of proinflammatory cytokines TNFα, IL-1α, IL-1β, IL-4, MIP-1α and MIP-1β were determined in c-Kit^+^ (b) and total bone marrow (d). Expression of G-CSF, IL-6, SCF, IL-3, Angpt-2 and GM-CSF were determined in total bone marrow (c). All C_t_ values were normalized to β-actin and *Shb* knockout samples were related to corresponding wild type values. Means are presented as 2^-ΔC^_t_ ± SEM to demonstrate fold change in mRNA content. Data are based on 6 mice of each genotype from 2 independent experiments for c-Kit^+^ cells and 3 mice of each genotype from 1 experiment for unfractionated bone marrow. Click here for file

Additional file 5: Figure S5 STAT5 activity in c-Kit^+^ bone marrow from leukemic mice. The activation of STAT5 was determined by Western blot analysis of tyrosine phosphorylation by immunoblotting for phospho- and total STAT5 respectively. Protein phosphorylation was related to total protein content on the same blot and signal strength was estimated by densitometric analysis. Means are presented in arbitrary units ± SEM and are based on 6 mice of each genotype in 2 independent experiments. Click here for file
