# Supplementary figures and images for: The Src homology-2 protein Shb modulates focal adhesion kinase signaling in a BCR-ABL myeloproliferative disorder causing accelerated progression of disease
Source: J Hematol Oncol. 2014 Jun 21;7:45. doi: 10.1186/1756-8722-7-45 (PMC4074852; doi:10.1186/1756-8722-7-45)

## Supplementary figure 1.

a

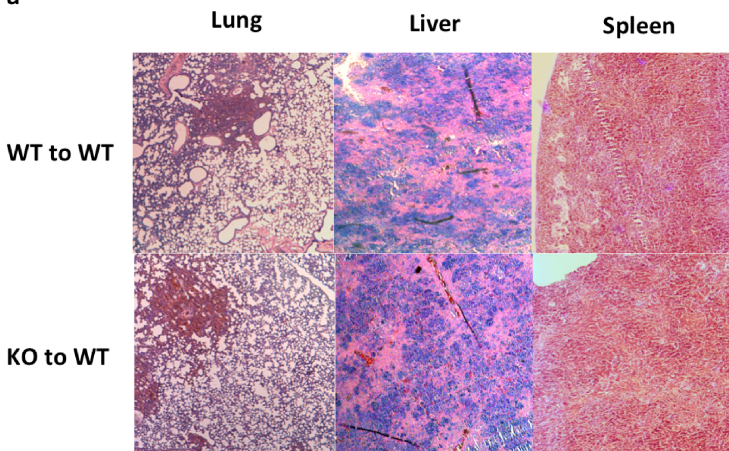

b

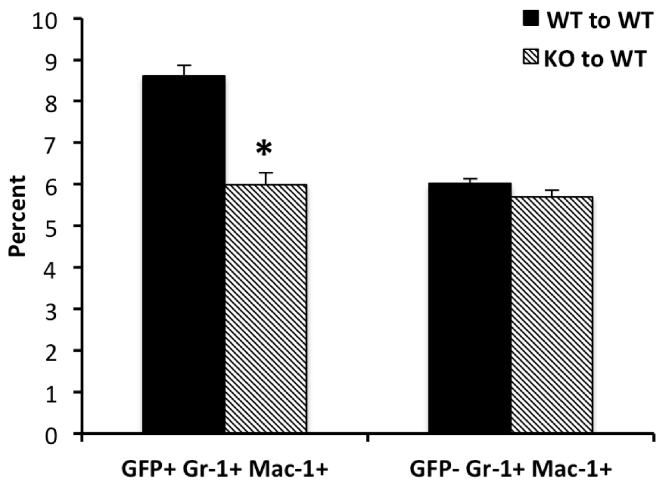

Supplement: Additional file 1: Figure S1 — A) Hematoxylin-eosin stained sections of lung, liver and spleen of diseased mice transplanted with BCR-ABL transformed bone marrow cells of wild type or Shb knockout background to wild type recipients. B) Percentage myeloid GFP + and GFP- cells in spleen. Means ± SEM are given and *indicates p < 0.05. [file 1756-8722-7-45-S1.pdf]

Supplementary figure 2.

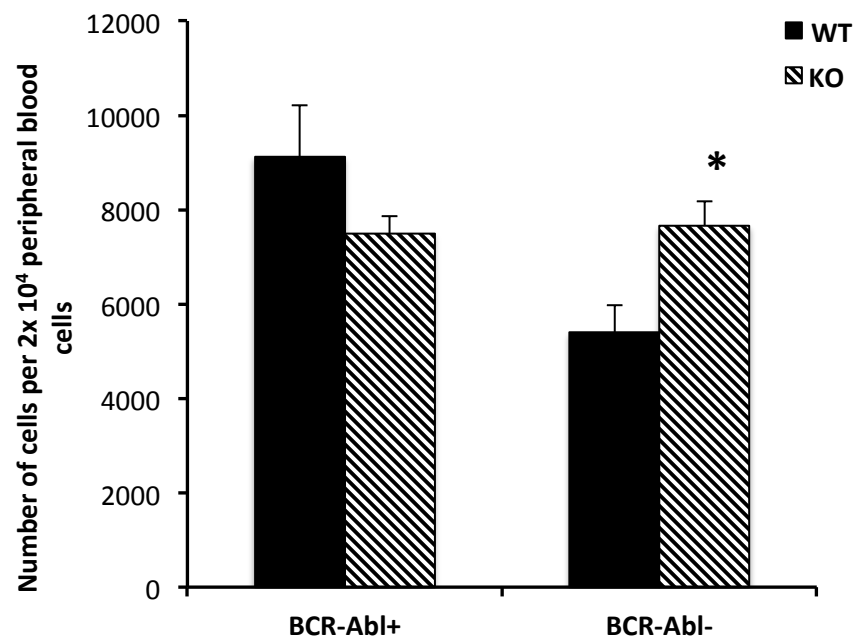

Supplement: Additional file 2: Figure S2 — Absolute numbers of BCR-ABL+GFP+ and BCR-ABL-GFP- myeloid cells in peripheral blood. GFP+ GR-1Hi Mac-1Hi and GFP- GR-1Hi Mac-1Hi cells were identified with FACS analysis in order to estimate the number of BCR-ABL carrying cells. Means are presented in arbitrary units ± SEM and are based on 6 mice of each genotype in 2 independent experiments. *denotes p < 0.05 as determined by Student’s t-test. [file 1756-8722-7-45-S2.pdf]

**Supplementary figure 3.**

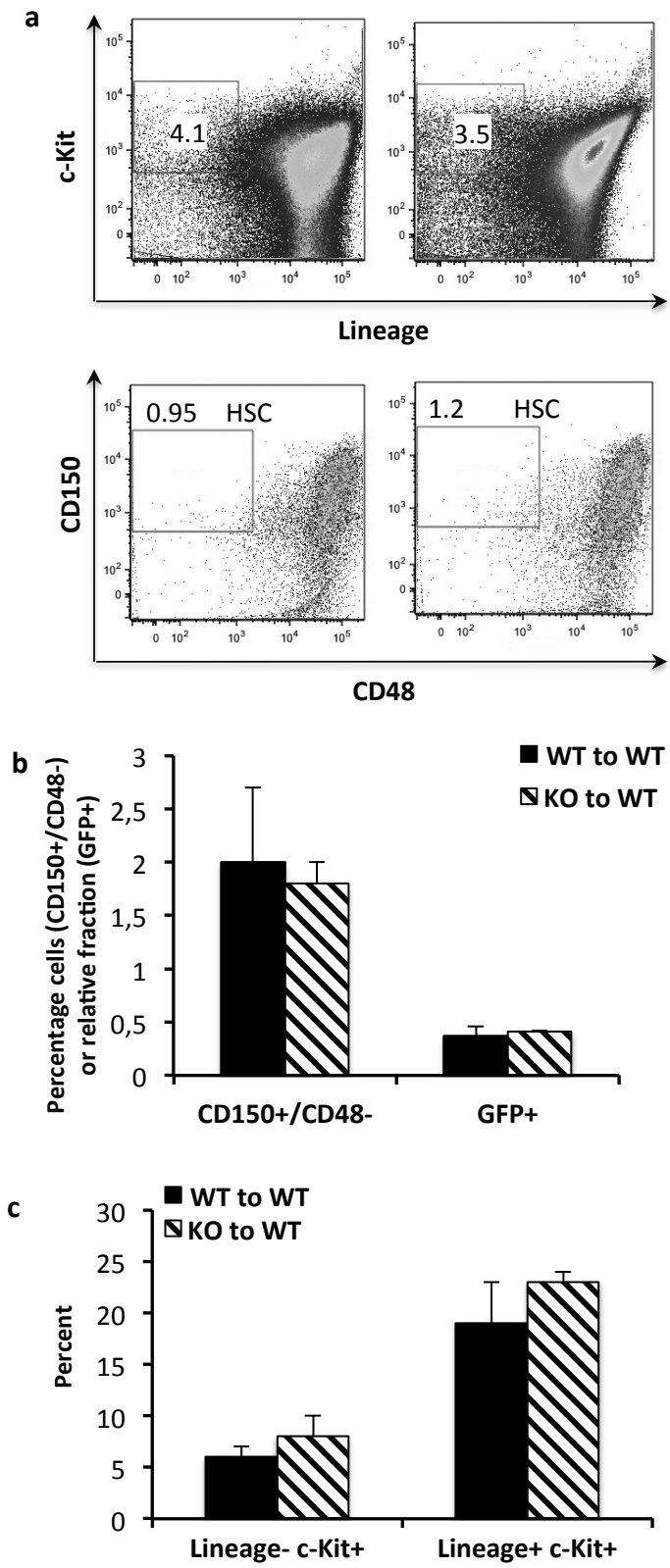

Supplement: Additional file 3: Figure S3 — Estimation of HSC proportions in murine BCR-ABL transformed bone marrow. The proportions of HSCs were established using FACS based on expression of lineage defining markers, c-Kit, CD48 and CD150 (a). Right panels are Shb knockout. (b) Percentage CD150+/CD48- cells among c-Kit+/Lin- cells and fraction of GFP + cells among the CD150+/CD48- cells. Means ± SEM for 6 experiments are shown. (c) Percentage lineage-/c-Kit + or lineage+/c-Kit + cells in bone marrows of wild type or Shb knockout BCR-ABL transformed bone marrow cells transplanted to wild type recipients. Means ± SEM for 6 mice of each genotype are given. [file 1756-8722-7-45-S3.pdf]

Supplementary figure 4.

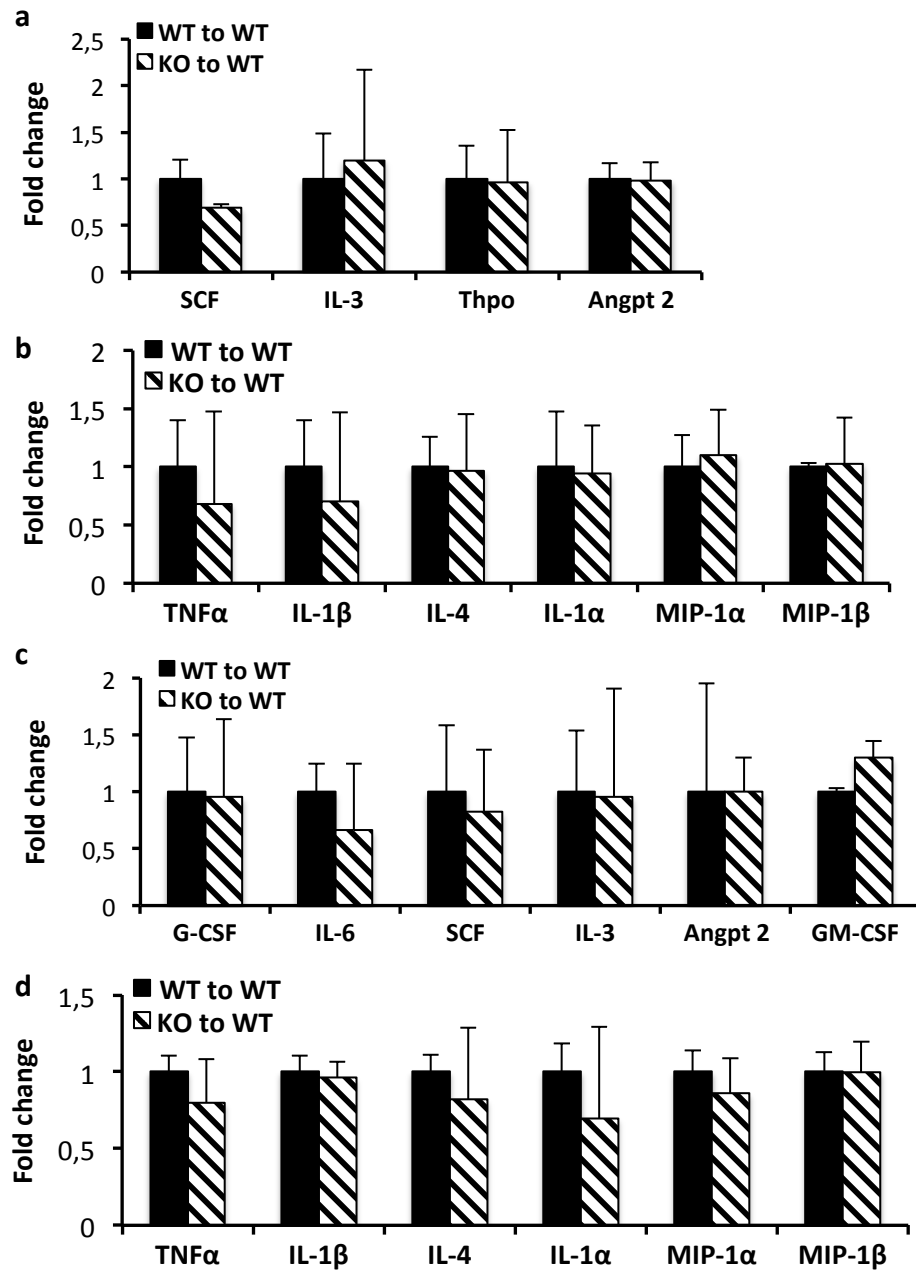

Supplement: Additional file 4: Figure S4 — Evaluation of cytokine expression in c-Kit enriched and unfractionated (total) bone marrow. (a) Transcript levels of SCF, IL-3, thrombopoietin (Thpo) and angiopoietin-2 (Angpt2) were evaluated with semi-quantitative real-time RT-PCR using mRNA isolated from c-Kit + leukemic bone marrow samples. The expression of proinflammatory cytokines TNFα, IL-1α, IL-1β, IL-4, MIP-1α and MIP-1β were determined in c-Kit+ (b) and total bone marrow (d). Expression of G-CSF, IL-6, SCF, IL-3, Angpt-2 and GM-CSF were determined in total bone marrow (c). All Ct values were normalized to β-actin and Shb knockout samples were related to corresponding wild type values. Means are presented as 2-ΔCt ± SEM to demonstrate fold change in mRNA content. Data are based on 6 mice of each genotype from 2 independent experiments for c-Kit+ cells and 3 mice of each genotype from 1 experiment for unfractionated bone marrow. [file 1756-8722-7-45-S4.pdf]

**Supplementary figure 5.**

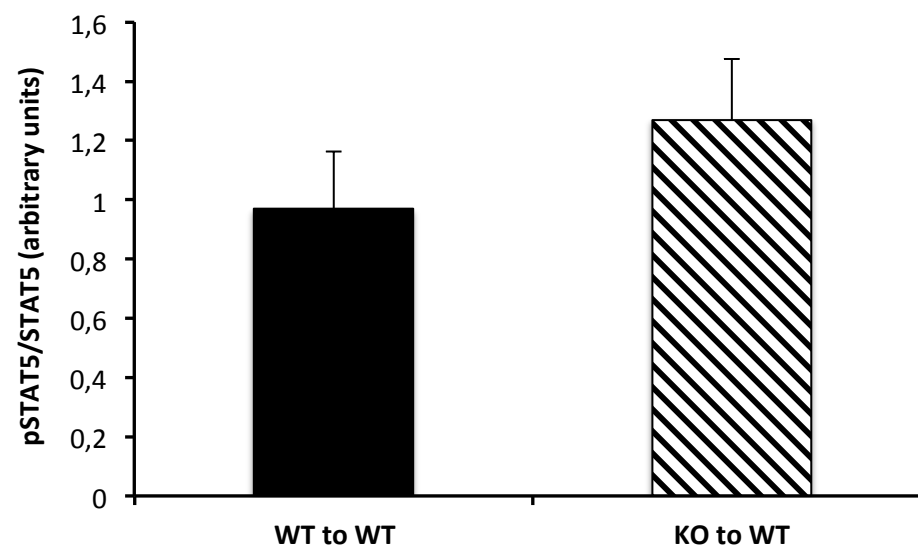

Supplement: Additional file 5: Figure S5 — STAT5 activity in c-Kit+ bone marrow from leukemic mice. The activation of STAT5 was determined by Western blot analysis of tyrosine phosphorylation by immunoblotting for phospho- and total STAT5 respectively. Protein phosphorylation was related to total protein content on the same blot and signal strength was estimated by densitometric analysis. Means are presented in arbitrary units ± SEM and are based on 6 mice of each genotype in 2 independent experiments. [file 1756-8722-7-45-S5.pdf]
